# Frankincense essential oil suppresses melanoma cancer through down regulation of Bcl-2/Bax cascade signaling and ameliorates heptotoxicity via phase I and II drug metabolizing enzymes

**DOI:** 10.18632/oncotarget.26930

**Published:** 2019-05-28

**Authors:** Faruck L. Hakkim, Hamid A. Bakshi, Shabia Khan, Mohamad Nasef, Rabia Farzand, Smitha Sam, Luay Rashan, Mohammed S. Al-Baloshi, Sidgi Syed Anwar Abdo Hasson, Ali Al Jabri, Paul A. McCarron, Murtaza M. Tambuwala

**Affiliations:** ^1^ Frankincense Biodiversity Unit, Research Center, Dhofar University, Salalah, Oman; ^2^ Department of Pharmacy, School of Applied Sciences, University of Huddersfield, Queensgate, Huddersfield, United Kingdom; ^3^ Department of Pathobiological Sciences, School of Veterinary Medicine, Louisiana State University, Baton Rouge, LA, USA; ^4^ Department of Clinical and Pharmaceutical Sciences, University of Hertfordshire, Hertfordshire, United Kingdom; ^5^ Chemotherapy Unit, St. Jude Clinics-Center for Cancer Treatment, Pathanamthitta, Kerala, India; ^6^ Department of Mathematics and Sciences, College of Arts and Applied Sciences, Dhofar University, Salalah, Oman; ^7^ Department of Microbiology and Immunology, College of Medicine and Health Sciences, Sultan Qaboos University, Al-Khoud, Muscat, Oman; ^8^ School of Pharmacy and Pharmaceutical Science, SAAD Centre for Pharmacy and Diabetes, Ulster University, Coleraine, County Londonderry, Northern Ireland, United Kingdom; ^*^ These authors contributed equally to this work and should be considered as first authors

**Keywords:** melanoma, frankincense, essential oil, apoptosis, tumor remission

## Abstract

Melanoma is a deadly form of malignancy and according to the World Health Organization 132,000 new cases of melanoma are diagnosed worldwide each year. Surgical resection and chemo/drug treatments opted for early and late stage of melanoma respectively, however detrimental post surgical and chemotherapy consequences are inevitable. Noticeably melanoma drug treatments are associated with liver injuries such as hepatitis and cholestasis which are very common. Alleviation of these clinical manifestations with better treatment options would enhance prognosis status and patients survival. Natural products which induce cytotoxicity with minimum side effects are of interest to achieve high therapeutic efficiency. In this study we investigated anti-melanoma and hepatoprotective activities of frankincense essential oil (FEO) in both *in vitro* and *in vivo* models. Pretreatment with FEO induce a significant (*p* < 0.05) dose-dependent reduction in the cell viability of mouse (B16-F10) and human melanoma (FM94) but not in the normal human epithelial melanocytes (HNEM). Immunoblot analysis showed that FEO induces down regulation of Bcl-2 and up regulation of BAX in B16-F10 cells whereas in FM94 cells FEO induced dose-dependent cleavage of caspase 3, caspase 9 and PARP. Furthermore, FEO (10 μg/ml) treatment down regulated MCL1 in a time-dependent manner in FM94 cells. *In vivo* toxicity analysis reveals that weekly single dose of FEO (1200 mg/kg body weight) did not elicit detrimental effect on body weight during four weeks of experimental period. Histology of tissue sections also indicated that there were no observable histopathologic differences in the brain, heart, liver, and kidney compare to control groups. FEO (300 and 600 mg/kg body weight) treatments significantly reduced the tumor burden in C57BL/6 mice melanoma model. Acetaminophen (750 mg/kg body weight) was used to induce hepatic injury in Swiss albino mice. Pre treatment with FEO (250 and 500 mg/kg body weight) for seven days retained hematology (complete blood count), biochemical parameters (AST, ALT, ALK, total bilirubin, total protein, glucose, albumin/globulin ratio, cholesterol and triglyceride), and the level of phase I and II drug metabolizing enzymes (cytochrome P450, cytochromeb5, glutathione-S-transferase) which were obstructed by the administration of acetaminophen. Further liver histology showed that FEO treatments reversed the damages (central vein dilation, hemorrhage, and nuclei condensation) caused by acetaminophen. In conclusion, FEO elicited marked anti-melanoma in both *in vitro* and *in vivo* with a significant heptoprotection.

## INTRODUCTION

Melanoma is one of the most aggressive forms of skin cancer with a high frequency of metastasis and a poor prognosis in the metastatic stage [1]. About 132,000 new cases of melanoma are diagnosed worldwide each year, according to the World Health Organization. Incidence rates are higher in women than in men before the age of 50, but by the age of 65, rates in men are double those in women, and by age 80 they are triple [2]. Despite advances in surgery and multi-agent chemotherapy, nearly 80% of patients still die from metastatic melanoma [3]. BRAF inhibitor and interleukin 2 therapy are often used in clinical set up but these regimens elicit severe toxicities with unsatisfactory efficacy and rapid development of resistance [4–6]. If patients were diagnosed early with primary melanoma, surgical resection is the best choice for most of them to reduce mortality [7]. However, a 5-year survival rate in metastatic melanoma is still under 15–20% of patients [8]. Clinicians prefer chemotherapy as a first line treatment option to avoid surgical consequences. High doses of chemotherapy are prescribed depending on the stage of melanoma but drug tolerance level of system is questionable. Conventional chemotherapy dosing schedule is used to balance the toxicity and efficacy, but the severe side effects in particular hepatotoxicity limits the administration of most anticancer agents [9]. Liver is the vital organ responsible for detoxification of drugs, however exposure of multiple doses of drugs leads to cholestatic hepatitis, progression to fibrosis and cirrhosis, malignant transformation, sinusoidal obstruction and fulminant hepatic failure [10–12]. Melanoma treatment is associated with liver injury. Ipilimumab and nivolumab well known target agents were approved by WHO to treat different stages of melanoma. Tanaka *et al.* reported that administration of both ipilimumab and nivolumab to 59 year old stage IV melanoma patients elicits severe hepatitis and elevation of liver injury associated serum biomarkers [13, 14]. This evidence alarms the urgent need of a safer alternative therapeutic regimen for melanoma.

Natural products (extracts or pure compounds) from various sources (plants, marine organisms, microorganisms, etc) are screened for their ability to act as anti-tumor agents and have shown to have structural diversity and chemical complexity, as well as modulating several cellular signaling pathways [15]. Reportedly, natural products activate anti-inflammatory, anti-tumor and/or anti-metastatic responses [15, 16] and also evade multidrug resistance [17, 18]. Vinblastine and paclitaxel, are well known anti cancer agents obtained from natural sources. Frankincense is an aromatic resin hardened from exuded gums obtained from trees of the genus *Boswellia* (*Burseraceae* family). *Boswellia* sp. includes *Boswellia sacra* from Oman and Yemen, *Boswellia carteri* from Somalia, and *Boswellia serrata* from India and China. It has been commonly used to reduce swelling and alleviate the pain of inflammatory diseases or tumors [19, 20]. Frankincense extract has been used in China for its accelerating effects on blood circulation. It has been used as an antiarthritic in ayuredic medicine in India for thousands of years [19]. Winking *et al.* reported that a frankincense extract induces apoptosis and prolong survival in a rat glioma model [21]. A methanol extract of *Boswellia serrata* inhibits abnormal skin cell proliferation induced by 12-O-tetradecanoylphorbol-13-acetate (TPA) and tumor promotion initiated by 7,12-dimethylbenz[a]anthracene (DMBA) in a mouse model [22]. In a human clinical study, a *Boswellia serrata* resin extract has been shown to reduce cerebral edema and potential anti-cancer activity in patients irradiated for brain tumors [23]. *Boswellia* sp. essential oil, an extract prepared by distillation of frankincense gum resins, is one of the most commonly used essential oils in aromatherapy and it is reported as potent anti-cancer agent in breast and pancreatic cancer models [24, 25]. Previously, we reported an anti-cancer activity induced by heavy terpenes extracted from frankincense in breast cancer model [26]. These evidences strongly suggests the anti-tumor potential of frankincense, however, the efficacy of frankincense essential oil on melanoma is not yet reported. In this study we assessed the effect of frankincense essential oil on *in vitro* and *in vivo* model of melanoma. Further experiments were also carried out to investigate its potential hepatoprotective efficacy.

## RESULTS

### Cell viability

**Figure 1:**
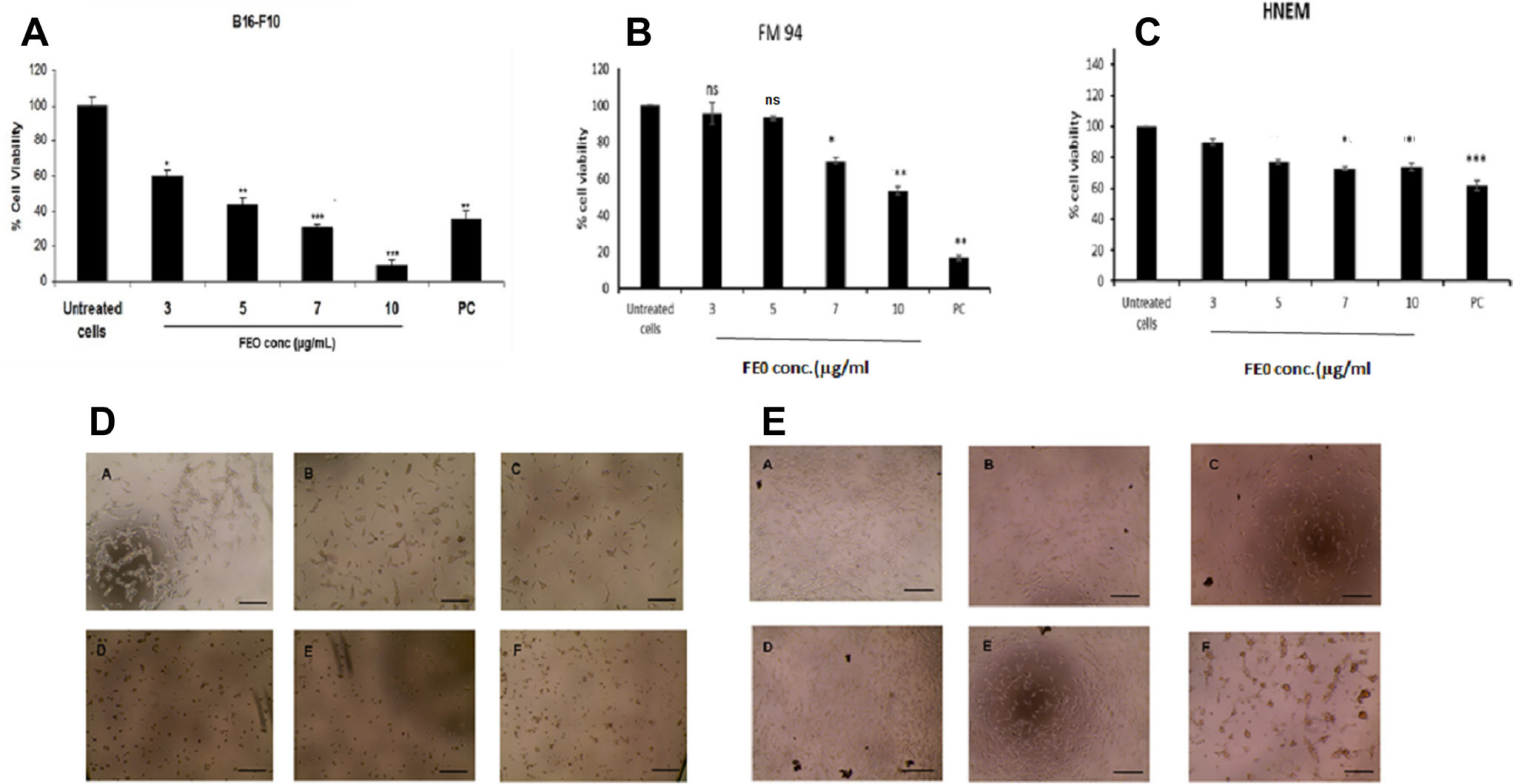
Cytotoxicity of FEO on B16-F10, FM94 and HNEM cells. (**A**) B16-F10 cells; (**B**) FM94 cells; (**C**) HNEM cells; (**D**) FM94 cells treated with FEO (A-Untreated cells; B-3 μg/ml; C-5 μg/ml; D-7 μg/ml; E-10 μg/ml; F- Dox 5 ug/ml) for 24 h and morphological image was photographed by EVOS image analyser; E: HNEM cells treated with FEO (A-Untreated cells; B-3 μg/ml; C-5 μg/ml; D-7 μg/ml; E-10 μg/ml; F- Dox 5 ug/ml) for 24 h and morphological image was photographed by EVOS image analyser. Data presented as mean ± SD of triplicates of three independent experiments. ^*^Represents significant difference at *p* < 0.05 compared with control. ^**^Represents significant difference at *p* < 0.01 compared with control. ^***^Represents significant difference at *p* < 0.001 compared with control. NS: Non significant. Scale bar indicates 10 um.

B16-F10 and FM94 cells were treated with FEO at 3, 5, 7 and 10 μg/ml for 24 h. FEO induced reduction in the cell viability of B16-F10 and FM94 carcinoma cells as compare to the untreated control (Figure 1A and 1B) and not normal human epithelial melanocyte (HNEM) (Figure 1C). FEO treated FM94 cells completely lost their adherence and morphology whereas HNEM cells retained their morphology (Figure 1D and 1E). The data indicated that pre treatment with FEO induced cytotoxicity in tumor cells by sparing normal cells.

### Nuclear fragmentation

**Figure 2:**
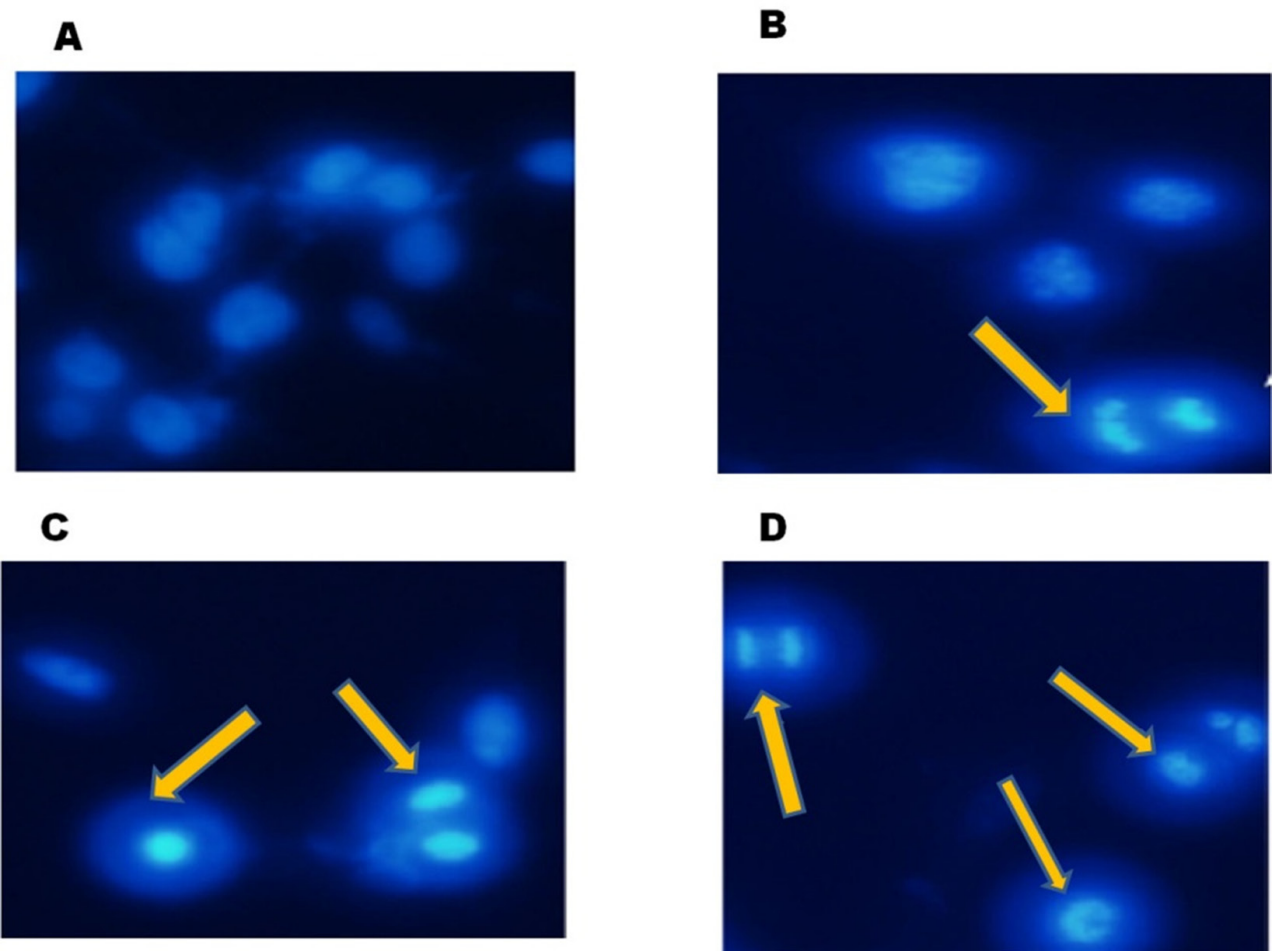
FEO induced nuclear fragmentation in B16-F10 cells: B16-F10 cells were treated with different concentrations of FEO for 24 hours and stained with hoechest stain 33258. (**A**) Control; (**B**) 5 ug/ml; (**C**) 7 ug/ml; (**D**) 10 ug/ml. The nuclei were observed using fluorescence microscope (20 um).

24 h of FEO treatment induced apoptosis in B16-F10 cells. Hoechest staining reveals that 5, 7 and 10 μg/ml of FEO induced nuclear fragmentation when compared to untreated control (Figure 2). These data indicate that FEO constituents target the nucleus of melanoma cells and cause DNA damage.

### DNA fragmentation

**Figure 3:**
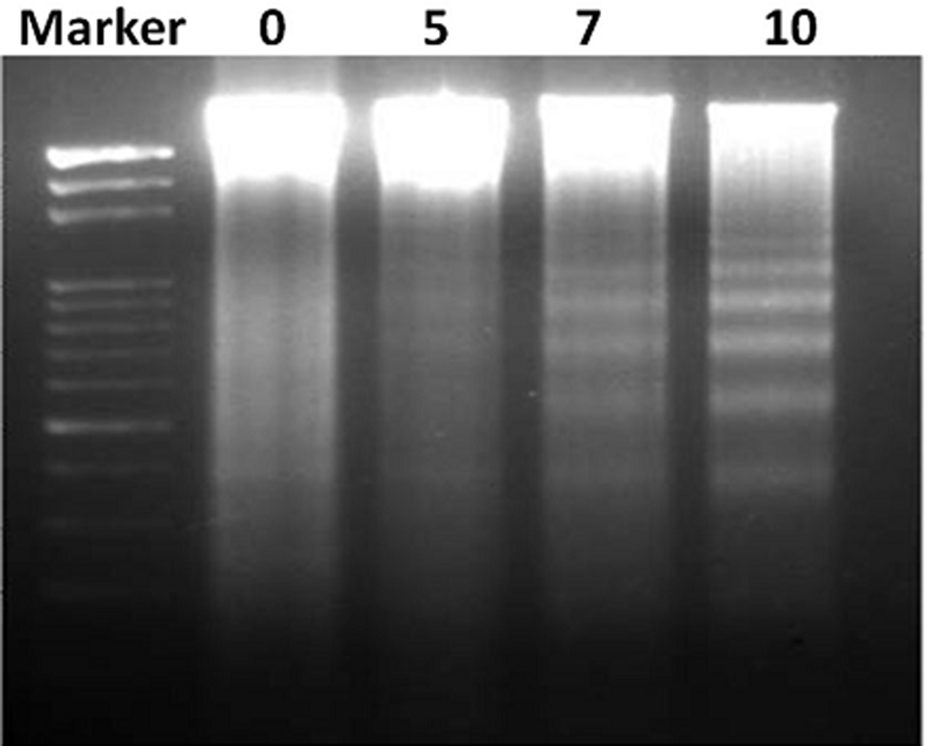
FEO induce DNA fragmentation in B16-F10 cells. B16-F10 cells were treated with 0, 5, 7 and 10 μg/ml for 24 hours. DNA was isolated resolved in agarose gel and examined by ethidium bromide staining.

To ensure the DNA targeting ability of FEO, B16-F10 cells were treated with different concentrations (5, 7 and 10 μg/ml) of FEO for 24 hrs and observed for DNA damage. Figure 3 shows that FEO induced DNA ladder formation in dose-dependent manner.

### Apoptosis

**Figure 4:**
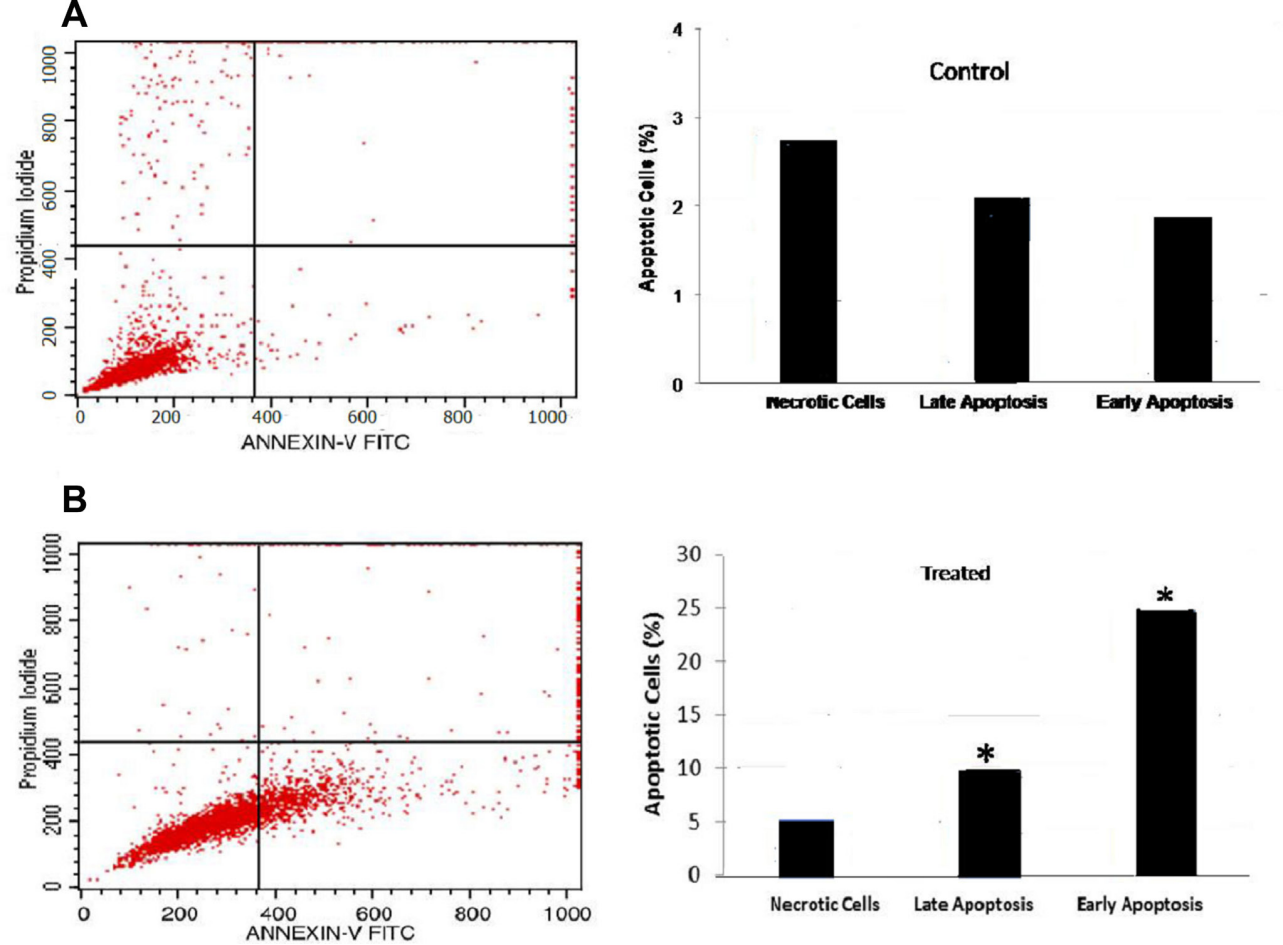
Flow cytometric analysis of apoptosis: B16-F10 cells treated with FEO at 10 μg/ml for 24 h. Annexin-V FITC and propidium iodide staining were used to analyze apoptosis by flow cytometry. (**A**) Untreated cells showed 2–3% of cells in early or late apoptosis stage. (**B**) FEO treated cells, significant increase (^*^P < 0.05) in percentage distribution of cells in early (25%) and late (10%) apoptosis were observed.

B16-F10 cells were treated with FEO at 10 μg/ml for 24 h and analyzed for apoptosis by annexin V staining using flow cytometry. FEO treatment resulted in a significant increase in percentage of cells in early (25%) and late (10%) apoptotic stage whereas only 2–3% of untreated cells reached early and late apoptosis stage (Figure 4).

### Molecular mechanism of inducing apoptosis by FEO

**Figure 5:**
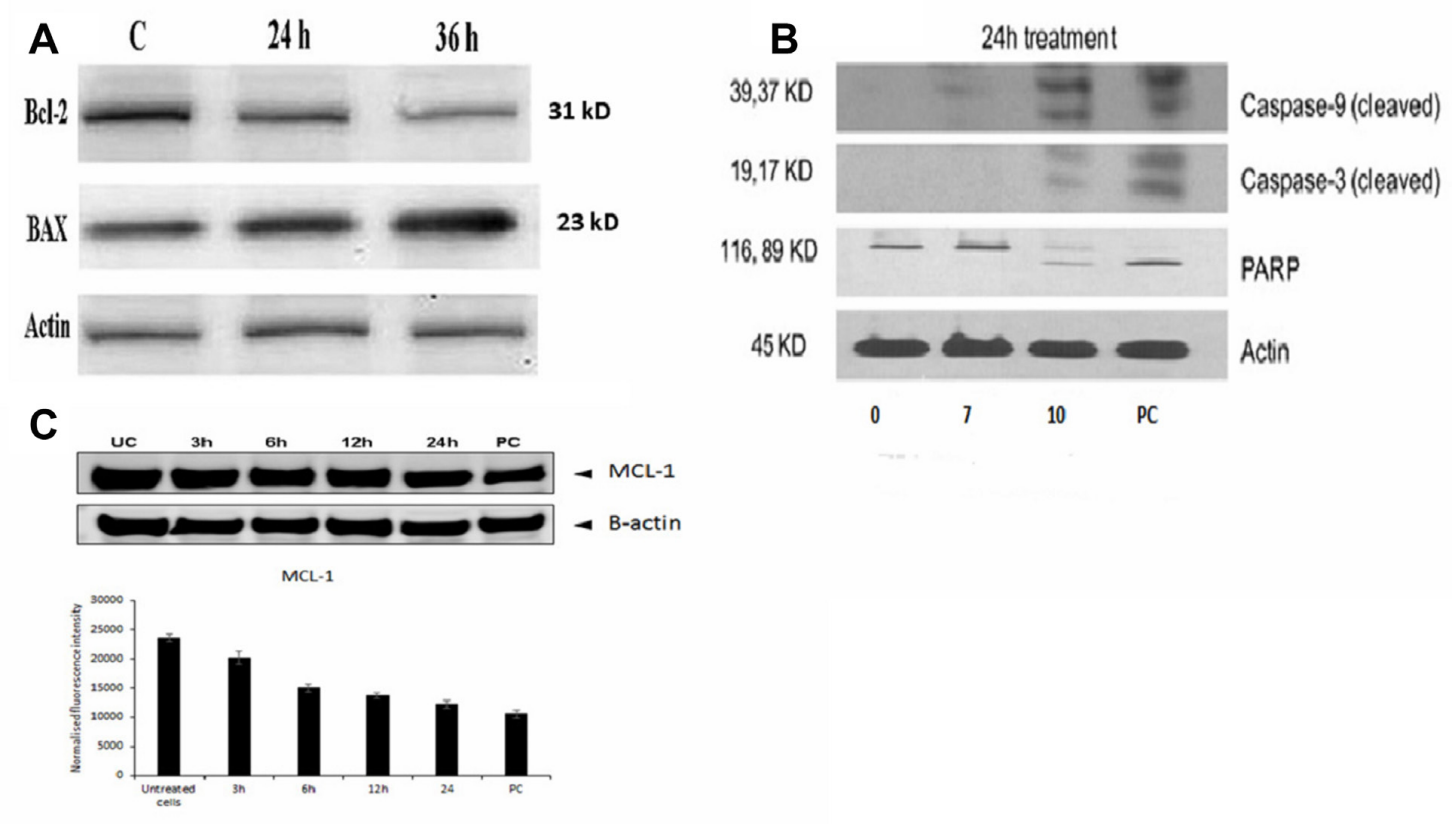
Molecular mechanism of FEO induced apoptosis in B16-F10 and FM94 cells. (**A**) Bcl-2 and Bax protein expression of B16-F10 cells was determined after 24 and 36 h of treatment with 10 μg/ml of FEO; (**B**) Cleaved Caspase 9, Caspase 3 and PARP expression of FM94 cells was determined after 24 h treatment with 0, 7, 10 μg/ml of FEO. PC indicates positive control (5 μg/ml of doxorubicin); (**C**) MCL-1 expression of FM94 cells was determined after 3 h, 6 h, 12 h, and 24 h treatment with 10 μg/ml of FEO. Actin used for normalization of protein expression.

To determine the mechanism of apoptotic induction in B16-F10 cells by FEO treatment, the expression levels of Bcl-2 and Bax by western blot were analyzed. The immunoblot analysis showed gradual decline in the expression levels of Bcl-2 with marked increase in the Bax protein expression levels in a time-dependent manner (Figure 5A). To investigate if the apoptosis was induced by FEO on human melanoma cells, FM94 cells treated with FEO (0, 7, 10 μg/ml) for 24 hours and the level of expression of the apoptotic markers (caspase 3, caspase 9, and PARP) were assessed. Figure 5B illustrates that FEO significantly up regulated the cleaved caspase 3, caspase 9, and PARP expression in dose-dependent manner. Further time-dependent down regulation of MCL-1 a major anti-apoptotic protein in melanoma was observed in FM94 cells by FEO (10 μg/ml) treatment (Figure 5C). This data suggest that FEO induced apoptosis in both mice B16-F10 and human FM94 carcinoma cells.

### *In vivo* toxicity experiment

**Figure 6:**
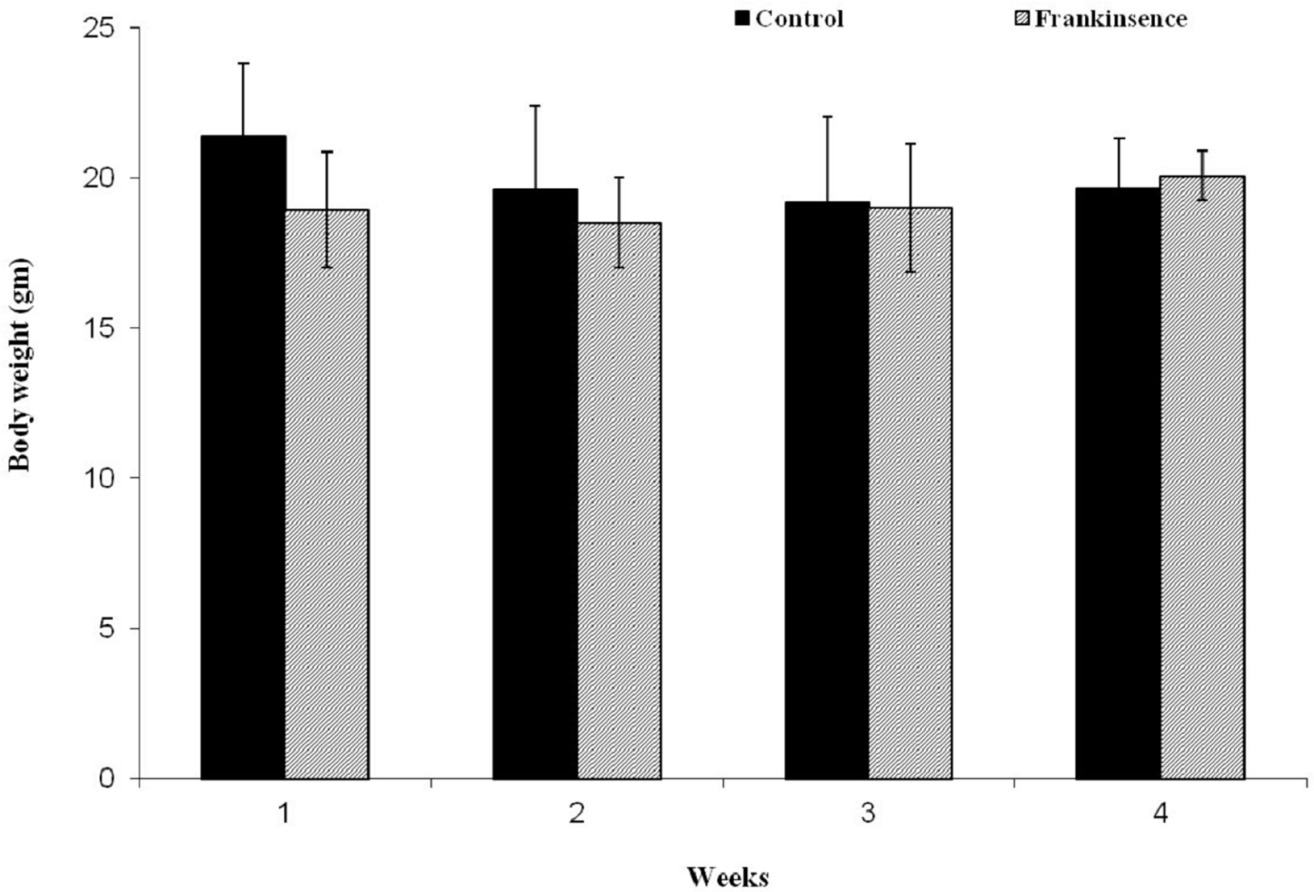
Effect of FEO on animal body weight: Data presented as mean ± SD (*n* = 8). Mice were treated with FEO (1200 mg/kg body weight) and observed for 30 days at weekly intervals.

**Figure 7:**
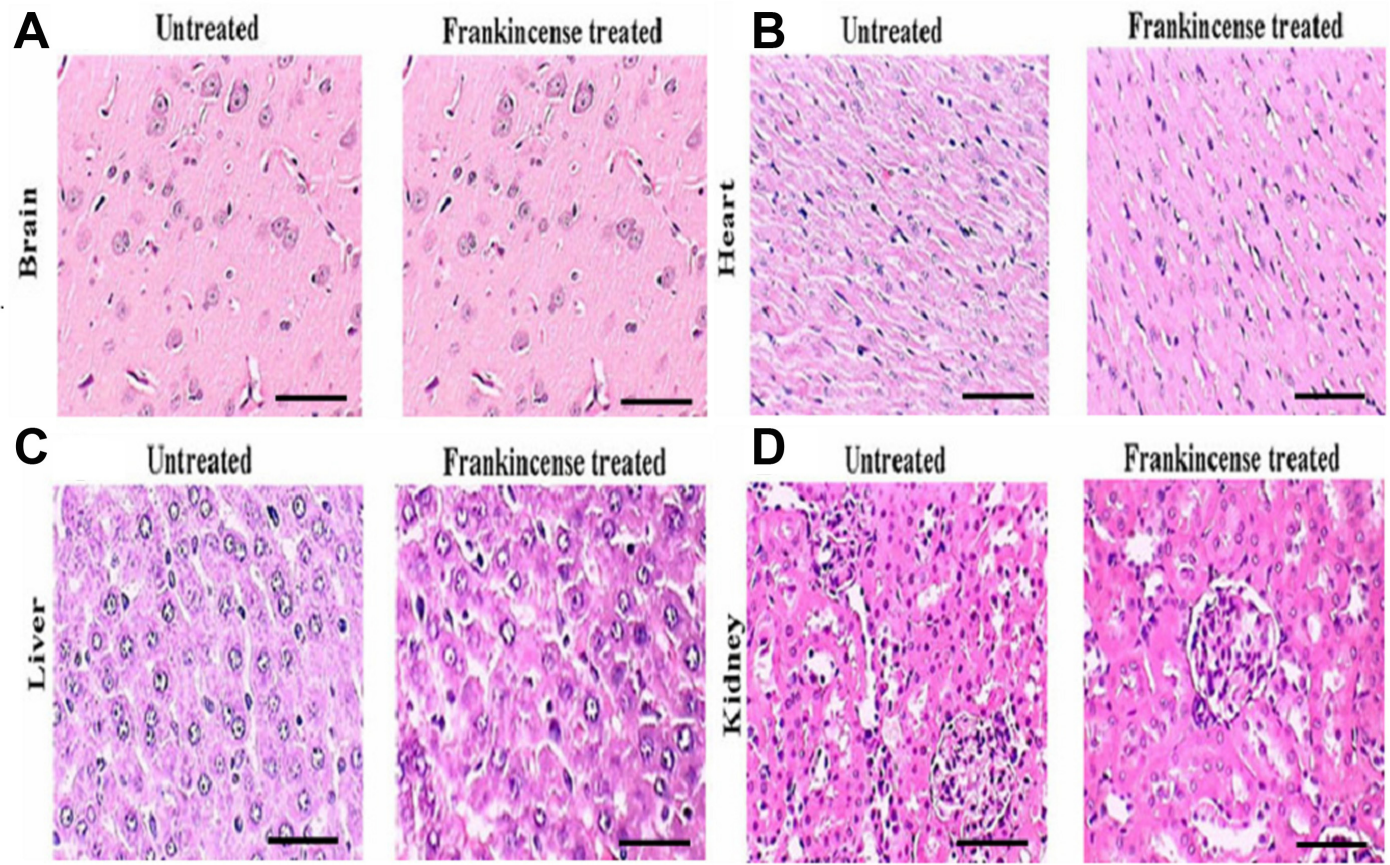
*In vivo* of toxicity of FEO on major organs: Mice were treated with FEO (1200 mg/kg body weight). At the end of experimental period major organs such as brain, heart, liver and kidney were excised and stained with hemotoxylin and eosin. Scale bar indicates 10 μm.

In order to assess the toxicity of FEO, mice were treated with a single dose of FEO (1200 mg/kg body weight) (i.p.) and observed for four weeks. Body weight and histology of major tissues (liver, kidney, brain, and heart) were analyzed. No significant difference in the body weight was observed in FEO treated mice compared to untreated control animals (Figure 6). Histological studies revealed that tissue sections from FEO-treated animals did not indicate any detectable pathologic abnormalities as examined by H&E staining. The liver showed normal hepatic lobular architecture, intact central vein with trapped red blood cells in a liver section from FEO treated animals. Kidney sections showed normal glomeruli, proximal and distal tubules, interstitium, and blood vessels. Brain and heart muscles normal morphology compared to untreated tissue sections (Figure 7).

### Tumour remission by FEO

**Figure 8:**
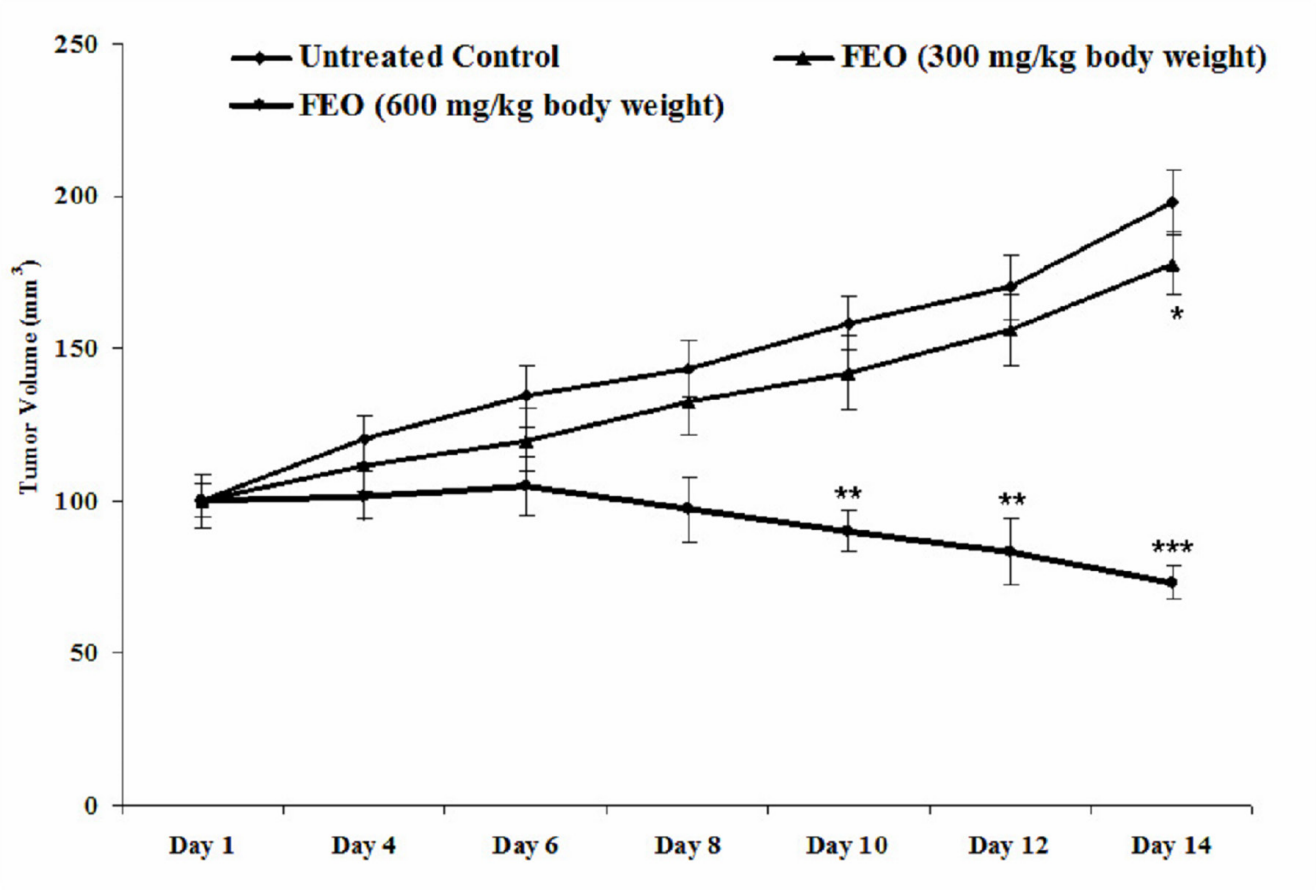
Melanoma tumor remission efficacy of FEO: Data presented as mean ± SD (*n* = 6). Tumor was induced by transplanting B16F10 cells (5 × 10^5^) in C57BL/6 mice. Tumor bearing mice treated with FEO (300 and 600 mg/kg of body weight) every two days for the total experimental period of 14 days and volume of tumor was measured. FEO 300 mg/kg bwt significantly (^*^p < 0.05) reduce the tumor volume on day 14 compare to control group. FEO 600 mg/kg bwt significantly (^**^p < 0.01, ^***^p < 0.001) reduced tumor burden on day 10, 12, and 14 compared to control group.

Melanoma tumour remission was observed in control and FEO treated tumour bearing mice. After 14 days the size of tumour was reduced in FEO treated mice compared to untreated control animals (Figure 8). Pattern of tumour growth inhibition revealed that FEO treatment reduced the tumor size dose dependently.

### *In vivo* hepatoprotective efficacy of FEO

#### Efficacy of FEO on hematology of acetaminophen induced hepatic injured mice

Acetaminophen is well known to induce both acute and chronic hepatic failure. Therefore in this study we used acetaminophen to develop hepatic injury mice model and assessed the efficacy of FEO on complete blood count (CBC). The PVC (35.20 ± 1.93), WBC (6100 ± 556.78), neutrophil (78.20 ± 0.58), Hb (6.07 ± 0.18), MCHC (24.1 ± 1.74), and RBC (6.40 ± 0.092), was declined and lymphocytes (20.00 ± 0.50), MCV (133.4 ± 23), MCH (31.1 ± 4.37) counts were significantly (*p* < 0.05) increased in acetaminophen (750 mg/kg body weight) treated mice. Acetaminophen induced abnormality was significantly (*p* < 0.05) reversed by FEO (250 and 500 mg/kg body weight) treatments (Table 1).

**Table 1:** Complete blood count: Values are expressed as mean ± SD (*n* = 8)

Parameters	Group I	Group II	Group III	Group IV
PVC	39.33 ± 0.67	35.20 ± 1.93	37.25 ± 3.04	33.75 ± 0.25
White blood cells (10 m/m^3^)	6500 ± 288.68	6100 ± 556.78	6500 ± 540.06	5000 ± 204.12
Lymphocytes	18.23 ± 1.76	20.00 ± 0.50^*^	20.40 ± 0.58	20.00 ± 0.82
Neutrophils	81.67 ± 1.76	78.20 ± 0.58^*^	79.50 ± 0.96	79.00 ± 0.86
Hb (g/dl)	8.10 ± 0.29	6.07 ± 0.18	6.93 ± 0.26	7.10 ± 0.70
MCV(μm^3^)	72.9 ± 7.97	133.4 ± 23	105.7 ± 15.6	88.0 ± 10.2
MCH (pg)	20.9 ± 2.21	31.1 ± 4.37	26.7 ± 4.04	22.7 ± 1.75
MCHC (%)	29.1 ± 1.88	24.1 ± 1.74	25.9 ± 2.31	26.7 ± 1.98
RBC (10^6^ /mm^3^)	8.67 ± 0.072	6.40 ± 0.092^*^	7.47 ± 0.0821^**^	8.52 ± 0.079

Group I: Normal control treated with saline (0.9%); Group II: Hepatotoxic control group treated with acetaminophen (750 mg/kg body weight); Group III: Treated with FEO (250 mg/kg body weight) and acetaminophen (750 mg/kg body weight); Group IV: Treated with FEO (500 mg/kg body weight) and acetaminophen (750 mg/kg body weight). ^*^Represents significant difference at *P* < 0.05 compared with control. ^**^Represents significant difference at *P* < 0.01 compared with hepatotoxic control group.

#### Efficacy of FEO on liver histology of acetaminophen induced hepatic injured mice

**Figure 9:**
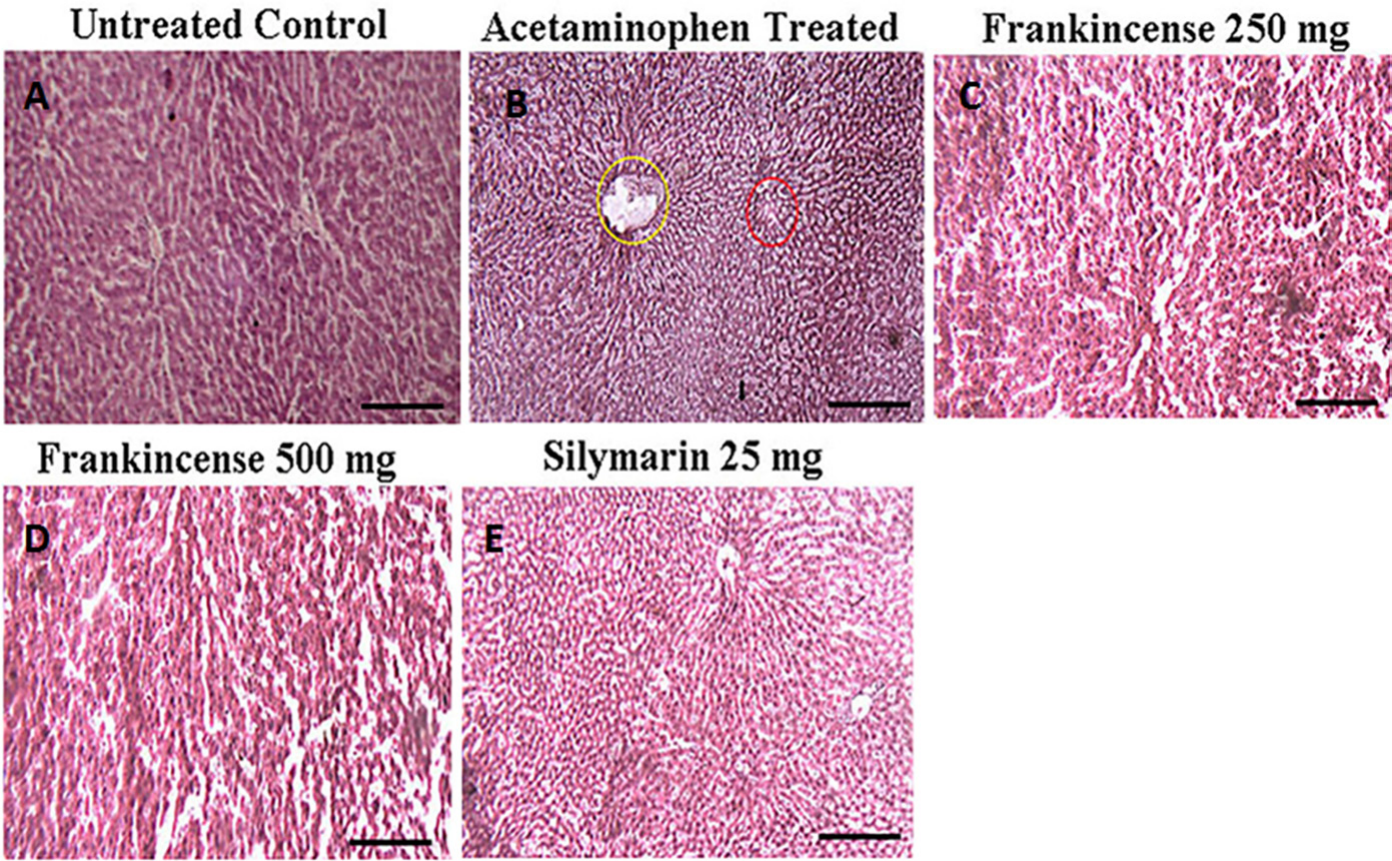
Efficacy of FEO on acetaminophen induced hepatic injury: Mice were divided into five groups (*n* = 6). (**A**) Untreated control: Mice received normal saline (0.9% v/w) i.p. for seven days. (**B**) Acetaminophen treated Acetaminophen (750 mg/kg body weight) alone injected to mice i.p. for seven days to induce hepatic injury. (**C**) Frankincense 250 mg: Mice treated simultaneously with Acetaminophen (750 mg/kg body weight) and FEO (250 mg/kg body weight) for seven days. (**D**) Frankincense 500 mg: Mice treated simultaneously with Acetaminophen (750 mg/kg body weight) and FEO (500 mg/kg body weight) for seven days. (**E**) Silymarin 25 mg: Mice treated simultaneously with Acetaminophen (750 mg/kg body weight) and silymarin (25 mg/kg body weight) (positive drug control). After seven days of treatment period the liver of all the animals was excised and histology was carried out. Scale bar indicates 10 μm.

Drug induced hepatic injury markedly disturb the morphology and arrangement of cells within liver tissue. Acetaminophen (750 mg/kg body weight) treatment induced mild sinusoidal congestion, hepatocellular necrosis, focal damage around the central vein, feathery degeneration, loss of lobular architecture with damaged cellular outlines, hemorrhage, nuclei have become condensed, and microvesicular fatty change (Figure 9). These detrimental effects of acetaminophen were restored by pre treatment with FEO (250 and 500 mg/kg body weight) and silymarin (25 mg/kg body weight).

#### Efficacy of FEO on biochemical parameters of acetaminophen induced hepatic injured mice

**Figure 10:**
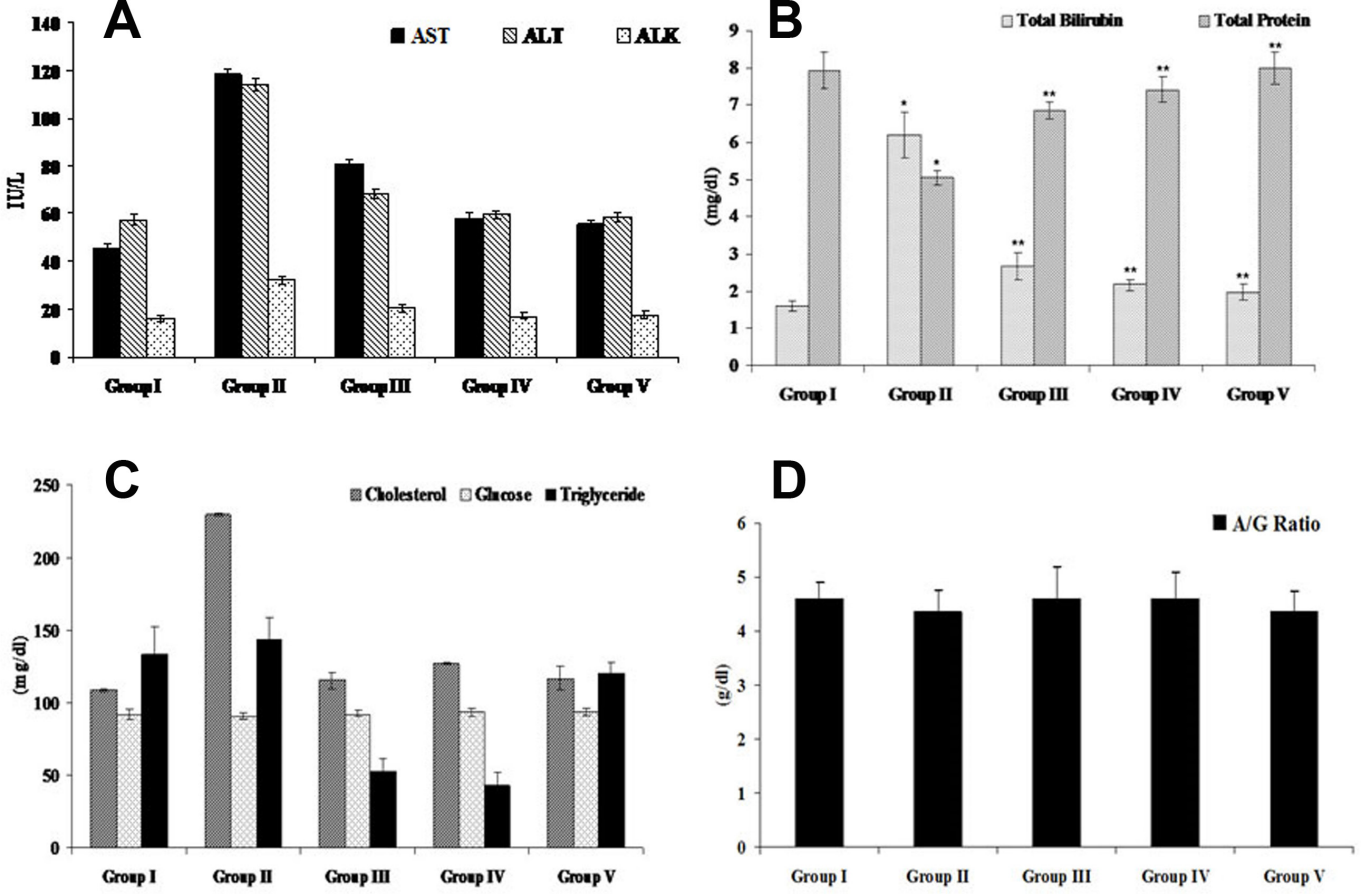
Efficacy of FEO on biochemical parameters of acetaminophen induced hepatic injured mice: Values are expressed as Mean ± SD (***n*** = 6). ^*^p < 0.05 is considered significant when compared with group I; ^**^p < 0.01 is considered significant when compared with group II by Dennett’s multiple comparison test. Group I: Untreated control fed orally with normal saline (0.9% v/w) 5ml/kg body weight daily. Group II as hepatotoxic control treated with acetaminophen (750 mg/kg body weight). Group III and IV received FEO 250 and 500 mg/kg body weight respectively along with acetaminophen (750 mg/kg body weight) for seven days. Group V treated with Silymarin (25 mg/kg body weight) with acetaminophen (750 mg/kg body weight) for seven days. (**A**) AST, ALT, ALK. (**B**) Total bilirubin and total protein. (**C**) Cholesterol, glucose, and triglyceride. (**D**) Albumin/globulin ratio.

Alteration in biochemical parameters is clinical manifestation of systemic toxicities including hepatotoxicity. In this study, we measured the levels of toxicity associated biomarkers such as AST, ALT, ALK, bilirubin, protein, cholesterol, glucose, albumin/globulin ratio and triglycerides in serum. Acetaminophen (750 mg/kg body weight) treated mice showed elevated levels of AST (118.50 ± 1.72, *p* < 0.05), ALT (114.00 ±2.67, *p* < 0.05), ALK (32.24 ± 1.96, *p* < 0.05), bilirubin (6.20 ± 0.63, *p* < 0.05), cholesterol (229.67 ± 0.75) and triglyceride (144 ± 14.4) whereas, the levels of protein (5.06 ±0.20, *p* < 0.05), glucose (90.66 ± 2.45), and albumin/globulin ratio (4.36 ± 0.4) were decreased (Figure 10). Seven days of pre treatment with FEO (250 and 500 mg/kg body weight) reversed the changes caused by acetaminophen and brought back the level of biochemical parameters to near normal control.

#### Efficacy of FEO on Phase I and II drug metabolizing enzymes

**Figure 11:**
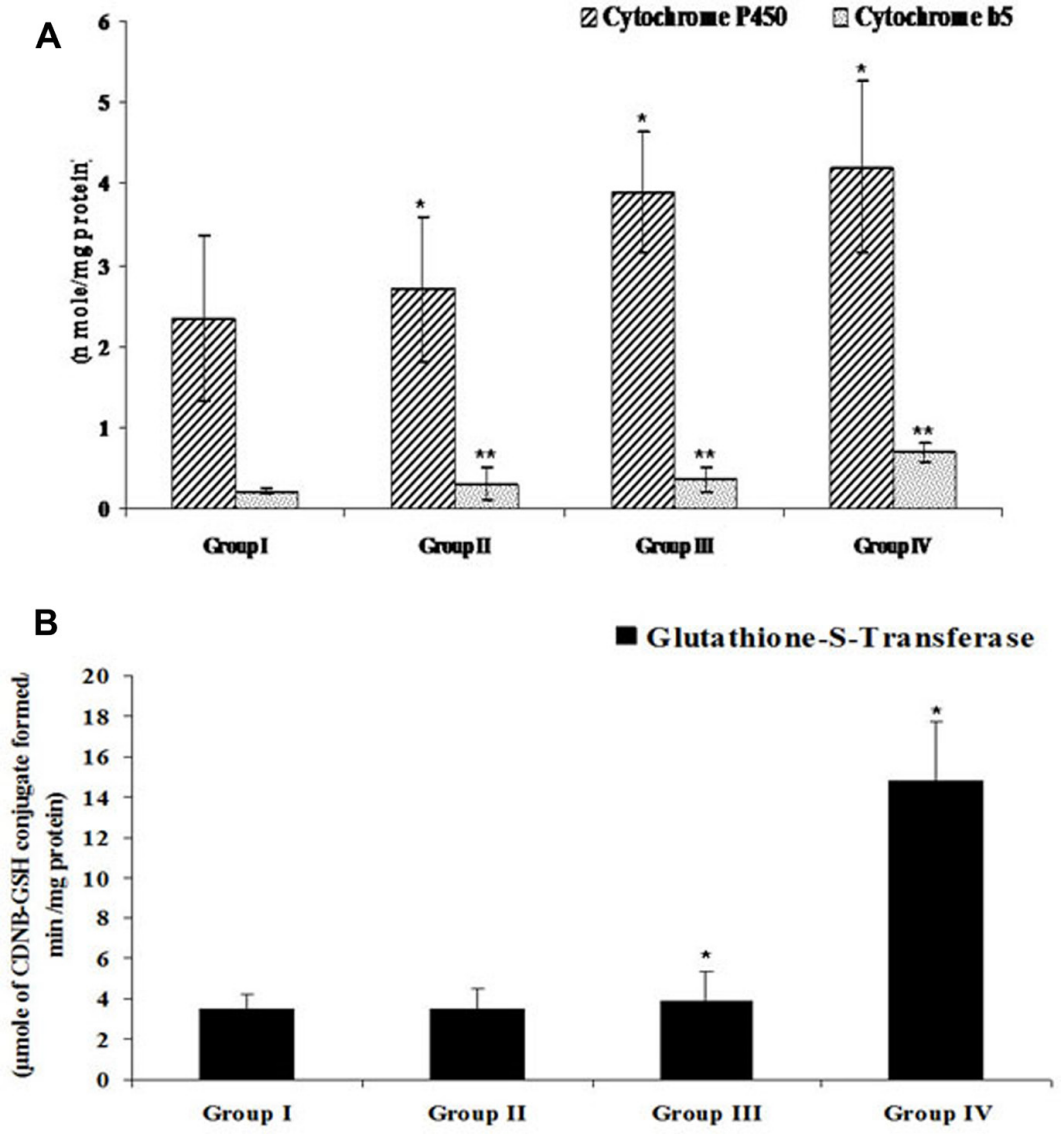
Efficacy of FEO on drug metabolizing enzymes of acetaminophen induced hepatic injured mice: Values are expressed as mean **±** SD (***n*** = 8) animals. ^*^p < 0.05, ^**^p < 0.01 represent significant changes against control. Group I: Untreated control and fed orally with distilled water daily for seven days. Group II: Mice treated with FEO 250 mg/kg body weight. Group III: Mice treated with FEO 500 mg/kg body. Group IV: Mice treated with 0.75% BHA along with diet (positive control). (**A**) CytP450 and Cytb5, (**B**) GST. BHA – butylated hydroxyanisole; Cyt P450 – Cytochrome P450; Cyt b5 – Cytochrome b5; GST – Glutathione S-transferase.

Drug metabolizing enzymes such as cytochrome p450 (Cytp450), cytochrome b5 (Cytb5) and glutathione-s-transferase (GST) play pivotal role in detoxification of drugs. To develop FEO based drug formulation for melanoma treatment it is imperative to study efficacy of FEO on above mentioned enzymes. Administration of 250 and 500 mg/kg body weight of FEO for seven days changed the levels of Cytp450 (2.7 ± 0.89, *p* < 0.001; 3.9 ± 0.74, *p* < 0.001 respectively), Cytb5 (0.31 ± 0.19 *p* < 0.01; 0.36 ± 0.14, *p* < 0.01 respectively), and GST (3.46 ± 1.08, *p* < 0.001; 3.89 ± 1.45, *p* < 0.001) significantly (Figure 11). Interestingly, treatment with FEO enhanced levels of these enzymes and this data clearly stated that FEO can retain hepatic physiological functions in particular detoxification. BHA was used positive diet control.

## DISCUSSION

Surgical resection for early stage and drug (Ipilimumab and nivolumab) therapies for advanced stage of melanoma (metastasis) are often chosen by the oncologist but poor prognosis status and a decline in the patient’s survival accompanied with post treatment toxicities are major concerns. Diagnosis at early stage and developing tolerable agents for therapy are major challenges. Thus natural products have attracted attention in recent years for developing effective and safe anti-cancer drugs. About 36% of the low molecular weight compounds approved by the Food and Drug Administration (FDA) are natural products or their derivatives [27]. Epidemiological studies states that natural products including resveratrol, lycopene, dioscin and polyunsaturated omega-3 fatty acids (PUFA), play an indispensable role in preventing cancers with lower toxicities [28–32]. Frankincense is well known plant in Middle Eastern regions (Yemen and Oman), India and China. Since ancient times it is a common practice that inhaling of frankincense resin smoke, drinking of aqueous extract of frankincense resin and chewing fresh resin for healing various ailments. Different varieties of frankincense are available and some of them are edible too. In this study, we used hojari variety of frankincense resin (*Boswellia sacra*) which is edible and it is consumed by local people. Frankincense extract has been studied for various biological activities in particular as an anti-cancer agent and different forms of boswellic acids have been isolated as it is principal components [33]. The use of frankincense essential oil (FEO) in aromatherapy is well known and it is also reported its potential as anti-cancer agent against breast and pancreatic cancer *in vitro* [24, 25]^.^ Despite the potential of FEO no concrete scientific evidence is available to translate this in to clinic. To explore further herein we report for the first time anti melanoma (*in vitro* and *in vivo*) efficacy and hepatoprotective capacity of FEO.

Cytotoxicity of FEO was tested in B16-F10, FM94 and HNEM cells. 24 h treatment with FEO significantly reduced the cell viability of B16-F10 and FM94 cells by sparing HNEM cells (Figure 1A–1C). This clearly indicates that FEO selectivity inducing cytotoxicity in carcinoma cells and not in normal cells. To unravel the mechanism FEO mediated cell death, FEO treated cells were subjected to apoptotic analysis. Biochemical signatures accompanied with apoptosis include chromosomal DNA cleavage into inter-nucleosomal fragments, phosphatidylserine externalization and a number of intracellular substrate cleavages by specific proteolysis [34]. Hoechest 33258 staining reveals that FEO treatment induced dose dependent nuclear damage in B16-F10 cells (Figure 2). Further experiment, indicated that FEO induces DNA damage in a dose dependent manner (Figure 3). It is well established that the principal component of FEO is α-pinene with other terpene mixtures [35]. These complex terpenes mixtures could intercalate with DNA to form adducts. Similarly α-pinene isolated from *Schinus terebinthifolius Raddi* induced DNA fragmentation in B16-F10 cells [36]. Previous works demonstrated that essential oils and other plant derived compounds can induce genomic DNA fragmentation in apoptotic cells [37, 38]. Though FEO treatment targeted the DNA in B16-F10 cells, it is imperative to understand apoptotic state of B16-F10 cells upon FEO treatment. Flow cytometric cell cycle analysis showed that FEO facilitates early apoptosis in B16-F10 cells than late apoptosis compared to untreated cells (Figure 4). Nuclear condensation and endonuclease G also translocates to the nucleus where it cleaves nuclear chromatin to produce oligonucleosomal DNA fragments which is referred to as early form of apoptosis [39, 40]. This is in agreement with our results where we observed FEO elicits extensive DNA damage with nuclear fragmentation. Induction of apoptosis is considered as a predominant therapeutic approach for various bioactive agents and natural products. Previous studies have indicated that Bcl-2 family proteins and pro-apoptotic proteins (Bax and Bak) play an important role in the intrinsic apoptotic pathway by regulation of mitochondria integrity [41]. While reduced Bcl-2 expression accompanied with high expression of Bax may promote apoptotic response to anticancer drugs, increased expression of Bcl-2 leads to resistance to chemotherapeutic drugs [42]. In our study, we found that FEO down regulates the expression of Bcl-2 with simultaneous up regulation of pro apoptotic Bax in B16-F10 cells (Figure 5A). FEO elicits apoptosis by down regulating the Bcl-2 which leads to formation of apoptosome in the cytosol and initiation of caspase signaling cascade.

In order to evaluate the molecular mechanism of FEO induced apoptosis, we treated human melanoma cells (FM94) with FEO and assessed caspase 3, caspase 9 and PARP expression. The hallmark of apoptosis is upon receiving death signal in the form of ligands (Fas, Death receptor 3, 4, 5, and TRAIL) or drugs, proapoptotic proteins such as caspase 3, 8 and 9 undergo post translational modifications that include dephosphorylation and cleavage leading to their activation and formation of death inducing signaling complex and translocated in to mitochondria where apoptosis initiated [43]. Moreover caspase-3 activation is responsible for cleaving most apoptotic substrates such as poly-(ADP-ribose) polymerase (PARP) [44, 45]. In this study we observed profound expression of cleaved caspase 3, caspase 9 and PARP in FM94 cells upon FEO treatment (Figure 5B). Our data indicates that induction of apoptosis by FEO was caspase-dependent and appeared to be mediated by the mitochondrial pathway. Similarly centipedegrass extract and docetaxel induced apoptosis in melanoma cells via caspase signaling [46, 47]. Studies found that MCL-1 major anti-apoptotic protein was frequently overexpressed and identified as an important target in majority of human cancers [48]. MCL-1 is known to be critical for survival of melanoma cells under various stress conditions [49, 50]. The expression of MCL-1 is also known to increase in melanoma with disease progression [51]. Reduction of MCL-1 expression weakens the link between pro- and anti-apoptotic Bcl-2 family proteins, such as Noxa/Mcl-1 and Bim/Mcl-1, resulting in the release of pro-apoptotic Bcl-2 family proteins. Thus Mcl-1 plays a unique role in regulating apoptosis, as elimination of Mcl-1 is required at an early stage for induction of apoptosis [49, 50] Therefore we studied the efficacy of FEO on MCL-1 expression in FM94 cells. We found that FEO down regulates MCL-1 time dependently (Figure 5C). This suggest a key role for MCL-1 in regulating apoptosis in FM94 cells induced by FEO. Similarly demethylzeylasteral and 9, 11-dehydroergosterol peroxide induce apoptosis in melanoma cells by down regulating MCL-1 [52, 53].

Toxicity is a limitation of chemotherapy therefore the search for safer and system tolerated agents is always encouraged by the WHO. To determine whether 1200 mg/kg body weight of FEO treatment elicits changes in body weight and toxicities to normal major organs, we examined brain, heart, liver and kidney of treated and untreated groups of Swiss albino mice. Histological sections of these tissues from FEO treated mice did not show any detectable pathologic abnormalities as examined by H&E staining (Figure 7). The mouse melanoma cell B16F10, derived from C57BL/6 mice, is one of the most malignant cancers in mice and can serve as a good model of human melanoma to evaluate various treatments including vaccines [54–56]. Therefore in this study we used melanoma xenograft model by injecting B16F-10 cells in C57BL/6 mice. FEO (300 and 600 mg/kg body weight) treatment reduced tumor size significantly compared to untreated control animals (Figure 8). This is in agreement with our *in vitro* data. Generally essential oils are usually devoid of long term genotoxic risks and many of them show antimutagenic capacity that can be linked to anticarcinogenic activity. Furthermore, studies have demonstrated that essential oil constituents are very efficient in reducing the melanoma tumour volume or tumour cell proliferation by exerting apoptotic effects [57, 58].

Chemotherapy induced liver injury can cause necrosis, steatosis, fibrosis, cholestasis, and vascular injury [59]. Stage IV melanoma patient treated with ipilimumab and nivolumab exhibit elevation of hepatic injury biomarkers [13, 14]^.^ Drug hepatotoxicity, is the leading cause of acute liver failure in the clinic worldwide [60]. *Boswellia sacra* extract shown to be hepatoprotective agent [61] however, FEO has not yet been reported for hepatoprotection effect. Acetaminophen is a classical hepatotoxin that is responsible for almost 50% of all acute liver failure cases in the USA, the UK and many Western countries [60]. Hepatotoxicity is a direct liver injury caused by the toxic metabolite of acetaminophen [62]. In this study, acetaminophen (750 mg/kg body weight) was used to induce hepatic injury. Hepatoprotective efficacy of FEO (250 and 500 mg/kg body weight) was studied on hepatic injured mice by assessing hematology (complete blood count), liver tissue histology, serum biochemical parameters, and phase I and II drug metabolizing enzymes. The analysis of blood cell count and Hb concentration are preliminary parameters to indicate abnormality. Pre treatment with acetaminophen altered the PVC, WBC, Lymphocytes, Neutrophils, Hb, MCV, MCH, MCHC%, and RBC. The administration of FEO reversed the altered counts in to normal level (Table 1). Our results evidenced that FEO can protect hepatic injury and associated blood cells abnormalities. Heavy dose of cancer drugs weakens the immune cells such as WBC, lymphocytes, and neutrophils which lead to their malfunction. A retrospective analysis of several ipilimumab Phase I–III trials in patients with advanced melanoma showed that immune related adverse events occurred in about 64.2% of treated patients and resulted in death [63]. Further Hb concentration along with RBC count determines the exchange of gases (O_2_ and CO_2_) to maintain homeostasis. FEO treatment restored blood cell counts and Hb level to normal values however; detail mechanistic study is required to delineate the role of FEO on blood components.

Levels of transaminases (AST and ALT) and liver histopathological and morphometrical changes were used as indicators of hepatotoxicity [64]. In this study we analyzed AST and ALT levels in acetaminophen along with FEO treated mice. Treatment with FEO (250 and 500 mg/kg body weight) reduced elevated levels of AST and ALT induced by acetaminophen administration (Figure 10). FEO could retain the liver structural integrity which was disturbed by acetaminophen because transaminases present in cytoplasmic location is released into the circulating blood only after structural damage [65, 66]. ALK is a marker of intra or extra hepatic cholestasis, its synthesis increases and the enzyme is thus released into plasma under such conditions. Drug-induced liver injury may present with a cholestatic pattern conjugated with hyperbilirubinemia accompanied by an increase in ALK levels [67]. In our study we found that treatment with acetaminophen enhanced the levels of serum ALK and bilirubin (Figure 10). Administration of FEO significantly reduced the ALK and bilirubin levels. Hepatotoxic agents induced biliary obstruction which is responsible for hyperbilirubinemia and enhanced ALK levels [68]. Our data indicates that treatment with FEO could protect the billiary obstruction caused by acetaminophen. Liver is the primary site of the synthesis of plasma proteins [69], disturbances of protein synthesis therefore occur as a consequence of impaired hepatic function which will lead to a decrease in the plasma concentrations. In our study we observed that acetaminophen treatment significantly reduced serum protein concentration. Seven days of treatment with FEO restored the total protein levels. This data shows that impairment of hepatic function caused by acetaminophen has been rescued by FEO. Albumin and globulin (A/G) accounts for the largest proportion of serum protein and they are generated by the liver and A/G ratio acting as one of the biomarkers for evaluating liver function [70]. In this study, we found that treatment with acetaminophen diminished the level of A/G ratio. However, treatment with FEO brought back the A/G ratio to normal levels **(**Figure 10**).** It was reported that clinical observation of decrease of serum albumin levels in hepatic diseases are associated with destruction or loss of parenchymal elements [71]. In hepatic diseases gamma globulin may be produced in increasing amounts by the hepatic reticuloendothelial cells [72] which eventually alters A/G ratio. Failure to return to normal should raise a suspicion of delayed recovery or chronic active hepatitis. Our data reveals that FEO might protect parenchymal and reticuloendothelial cells to balance the A/G ratio. High triglyceride and cholesterol level in hepatic injury is the clinical manifestation of cholestasis or hepatitis. Generally, the level of plasma lipids tends to decrease with the severity of liver disease [73, 74]. This is in agreement with our results, where we found acetaminophen (750 mg/kg body weight) increased the level of cholesterol and triglycerides (Figure 10). The results evidenced that acetaminophen might induce reduction in lipase activity, which could lead to decrease in triglyceride hydrolysis and damage of hepatic parenchymal cells that subsequently leads to disturbance of lipid metabolism in the liver. Administration of FEO (250 and 500 mg/kg body weight) diminished levels of cholesterol and triglyceride. These finding showed that mechanism of lipid lowering effects of FEO might be attributed to an inhibitory activity on microsomal acyl coenzyme A: cholesterol acyltransferase enzyme. This enzyme is responsible for acylation of cholesterol to cholesterol esters in the liver. Furthermore, level of glucose was measured in acetaminophen-induced hepatic injured mice since maintenance of blood glucose requires the relatively intact liver cells to release glucose and hepatic insufficiency. The latter might leads to production of several abnormalities directly or indirectly associated with carbohydrate metabolism [75]. In the present study administration of FEO maintained the level of glucose similar to control level (Figure 10). It is suggested that FEO might protect glucose releasing hepatocytes to maintain the constant/normal glucose level.

Histopathological study of liver tissue revealed that normal control group showed normal hepatic cells with intact cytoplasm and well defined nucleus with restricted number of inflammatory cells (Figure 9A); whereas acetaminophen treated group, showed a central vein dilated along with hemorrhage, nuclei condensation, cytoplasmic membrane destruction. In addition, scattered lymphocyte and plasma cells were also seen around portal triad (Figure 9B). The FEO and silymarin treated groups showed less damage with intact cytoplasm and nucleus and appeared to have normal hepatic cells (Figure 9C, 9D, and 9E). Phase I and Phase II drug metabolizing enzymes play an important role in converting toxic agents into non toxic and soluble substances. The rate limiting step in the activation and detoxification of chemical carcinogens and xenobiotics have been postulated to be dependent on the rate of reduction of cytochrome 450 (CytP450) substrate complex which in turn is dependent on the content of cytochrome b5 (Cytb5) and on the activity of NADPH cytochrome-c reductase [76]. Glutathione S-transferase (GST) is one of the detoxification systems in most human and animal tissues [77]. Organs with low levels GST are more susceptible to the toxic action of a variety of alkylating and therapeutic agents. In this study FEO (250 and 500 mg/kg body weight) treated mice showed increased levels of Cyt P450, Cyt b5, and GST (Figure 11). Therefore, it is suggested that FEO treatment mediated enhancement of Cyt P450, Cyt b5, and GST might act as a defense mechanism to protect the liver and probably other organs against the toxicity.

## MATERIALS AND METHODS

### *Boswellia sacra* essential oil preparation

Certified Hojari grade *Boswellia sacra* gum resins were obtained from the local market. Essential oil was extracted by hydrodistillation method as described earlier [24]. Briefly, *Boswellia sacra* resins were loaded into 55° C water with a ratio of 1:2.5 (w/v), and mixed with an electromechanical agitator for 30–45 min or until a thick homogenous mucilage was formed. Temperatures of the hydrodistiller were monitored by an infrared thermometer; and pressures were recorded at the condenser terminal. To remove any residual water, collected *Boswellia sacra* essential oil (FEO) was immediately transferred into a –20° C freezer; and ice crystals were separated from the essential oil.

### Cell lines and culture method

Mouse melanoma (B16-F10) cells were purchased from American Type Culture Collection, USA. Human melanoma cells (FM94) is kind gift from Dr. Sobia Kauser, University of Bradford, UK. Human normal epithelial melanocytes (HNEM) purchased from Promo cells, UK. B16-F10 and FM94 cells were cultured in Roswell Park Memorial Institute (RPMI 1640) medium with 10% fetal bovine serum and 1% antibiotics (penicillin/streptomycin) and HNEM cells were cultured in Promo cell melanocytes growth medium. Cells were maintained in humidified cell incubator at 37° C and 5% CO_2_.

### Drug preparation

Stock solution of FEO was prepared in dimethyl sulfoxide. Different concentrations of (3, 5, 7 and 10 μg/ml) were added in the cell culture medium.

### MTT assay

B16-F10, FM94, HNEM cells (1 × 105/well) were seeded in 96 well plates (100 μL/well) and allowed to adhere firmly overnight in respective medium with 10% fetal bovine serum. Then cells were treated with different concentrations (3, 5, 7 and 10 μg/ml) of FEO for 24 h. After 24h elapsed medium was removed and cells were incubated with MTT reagent (5 mg/mL) for 4 h and violet formazan crystals were dissolved in dimethylsulfoxide and absorbance was read at 540/690 nm. Absorbance of control (without treatment) was considered as 100% cell viability. Doxorubicin was used as positive drug control.

### Fluorescence microscopic analysis of apoptosis

B16-F10, 1 × 10^5^ cells/ml were seeded in 96 well plates (100 μ/well) and allowed to adhere firmly over night. The cells were then treated with different concentrations of FEO (5, 7 and 10 μg/ml). After 24 h treatment, 20 μl of trypsin was added into each well. Trypsinized cell suspension washed with phosphate buffered saline (PBS) and 25 μl cell suspension was transferred to glass slides. Hoechest 33258 (Sigma, St. Louis, MO, USA) staining solution was added to each suspension and then covered with a coverslip. The morphology of apoptotic cells was examined and visualized under a fluorescence microscope (Nikon Eclipse, Inc, Japan) at 400× magnification.

### DNA fragmentation by agarose gel electrophoresis

Inter-nucleosomal cleavage of DNA was analyzed as described previously [78]. B16-F10 cells were seeded in 6-well plates at a concentration of 1 × 10^6^ cells per ml of medium. Cells were then treated with FEO (5, 7 and 10 μg/ml). Cells were harvested after 24 hours of treatment and DNA fragmentation was assessed by gel electrophoresis.

### Annexin-V assay

B16-F10 (2 × 10 ^5^cells/10 cm dish) cells were treated with 10 μg/ml of FEO for 24 h. Then cells were harvested by centrifugation, washed with ice-cold PBS, and then resuspended with ice-cold 70% ethanol overnight. The following day, cells were treated with 10 μg/ml of RNase at 37° C, then spun down and stained with 40 μg/ml of propidium iodide (PI) and Annexin-V-FITC for 30 min. The apoptotic state of cells was measured by flow cytometry (FACS, BD Bioscience).

### Western blot analysis

The whole cell lysate was prepared from B16-F10 cells treated with FEO (10 μg/ml) for 24 and 36 hours, and FEO (0, 7, and 10 μg/ml) treated FM94 cells for 24 hours as described earlier [79]. Then whole cell lysate were resolved in a 10% SDS polyacrylamide gel electrophoretically and electro transferred onto a nitrocellulose membrane. The immunoblots were probed with Bcl-2, Bax, caspase 3 (cleaved), Caspase 9 (cleaved), and PARP antibodies and were visualized with the NBT/BCIP chromogenic substrate. Further MCL-1 expression was determined in FM94 cells treated with FEO at 10 μg/ml for 3, 6, 12, 24 hours as described above. Antibodies purchased from Cell signaling technology, UK.

### *In vivo* experiments

#### Animals

The swiss albino and C57BL/6 mice were selected from a random breed colony maintained in the animal house of Jawaharlal Nehru Cancer Hospital and Research Center, Bhopal, India. The mice were housed in polypropylene cages containing sterile paddy husk (procured locally) as bedding and maintained under controlled conditions of temperature (23 ± 20° C), humidity (50 ± 5%) and light and dark (10–14 h) respectively. The animals are fed with standard mice feed (formula obtained from Cancer Research institute, Mumbai, India) and filtered acidified water *ad libitum*. Mice of either sex, 6–8 weeks old and weighing 22 ± 2 g, were selected from the above colony for the experiments. The study was carried out as per OECD guidelines for testing for chemicals under IAEC number JNCHRC/13/IAEC/PN-185/B. All the experiments were conducted under the guidelines of Institutional Animal Ethical Committee (Project No.500/01/08/2016 /project 5 /28/07/2019) and confirms to the guidelines set by WHO (World health organizations, Geneva, Switzerland and INSA, New Delhi, India.

#### *In vivo* toxicity assessment

Swiss albino mice were divided in to two groups (*n* = 16). Group I treated with vehicle (saline) and group II treated with FEO (1200 mg/ kg of body weight) intraperitoneally (i.p.) every week of total four weeks. Body weight was measured for 30 days on weekly basis.

### Histopathological analysis

At the end of the experimental period, animals were euthanized by cervical dislocation and liver, kidney, brain, and heart tissues were dissected and examined by gross and microscopic anatomy. Then tissue sections were stained with hematoxylin and eosin staining as described earlier [80]. Microscopic evaluations were done by the pathologist.

### Induction of tumour (melanoma) in mice

Cell suspension of B16F10 cells (5 × 10^5^) was implanted in C57BL/6 mice subcutaneously at the shaved part. The mice bearing the tumour were randomly divided into 3 groups with 6 mice in each group. The dosing was started when tumour has reached a mean diameter of 100 mm^3^ and this day was designated as day 0.

### Experimental design

Mice (*n* = 18) were randomized into following three groups: Group I (Control (vehicle) treated with normal saline (0.9% v/w) i.p., Group II treated with FEO 300 mg/kg of body weight (i.p), and Group III treated with FEO 600 mg/kg of body weight (i.p) every two days for two weeks of experimental period. The tumour size was measured every alternate day using vernier calipers, and the tumour volume was calculated.

### Hepatoprotective activity

#### Hematological analysis

Animals were randomized and divided into four groups (*n* = 8). Group I served as untreated control (treated with normal saline (0.9% v/w) i.p. daily for seven days). Group II as hepatotoxic control treated with acetaminophen (750 mg/kg body weight) i.p. for seven days. Group III received acetaminophen (750 mg/kg body weight) and FEO (250 mg/kg body weight) concurrently i.p. for seven days. Group IV received acetaminophen (750 mg/kg body weight) and FEO (500 mg/kg body weight) concurrently i.p. for seven days. At the end of experimental period blood samples were collected via vein puncture into sterile sample tubes containing the anticoagulant, EDTA. Packed cell volume (PVC), White blood cells (WBC) count, Lymphocytes count, Neutrophils count, hemoglobin concentration (Hb), Mean corpuscular volume (MCV), Mean Corpuscular Hemoglobin (MCH), Percent of Mean Corpuscular Hemoglobin Concentration (MCHC%), red blood cell (RBC) were analysed using an automated analyser, Cell Dyne, model 331430, Abbott laboratories, IL, USA.

### Determination of biochemical parameters and histology of liver

Animals were randomized and divided into five groups (*n* = 8). Group I served as untreated control (treated with normal saline (0.9% v/w) i.p. daily for seven days). Group II as hepatotoxic control treated with acetaminophen (750 mg/kg body weight) i.p., for seven days. Group III received aacetaminophen (750 mg/kg body weight) and FEO (250 mg/kg body weight) concurrently i.p. for seven days. Group IV received aacetaminophen (750 mg/kg body weight) and FEO (500 mg/kg body weight) concurrently i.p. for seven days. Group V treated with acetaminophen (750 mg/kg body weight) and positive drug control Silymarin (25 mg/kg body weight) i.p., concurrently for seven days. At the end of experimental period blood samples were collected by puncturing retro-orbital plexus. The blood samples were allowed to clot for 45 min at room temperature. Serum was separated by centrifugation at 2500 rpm for 15 min at room temperature. Serum biochemical parameters such as serum glutamic oxaloacetic transaminase (AST), serum glutamic pyruvic transaminase, (ALT), serum alkaline phosphatase (ALK), bilirubin, protein, cholesterol, glucose, albumin/globulin ratio, triglyceride were measured as described earlier. Furthermore, liver was excised from animals and subjected to histological analysis by hematoxylin and eosin staining method.

### Determination of phase I and phase II drug metabolizing enzymes

Animals were randomized and divided into four groups (*n* = 8). Group I served as untreated control and fed orally with distilled water daily for seven days. Group II and III received FEO 250 and 500 mg/kg body weight (i.p), respectively for seven days. Group IV served as positive control and treated with 0.75% butylated hydroxyanisole (BHA) along with diet. At the end of experimental period livers were excised immediately and washed in ice cold normal saline, followed by 0.15 M Tris-Hcl (pH 7.4) blotted dry and weighed. A 10% (w/v) of homogenate was prepared in 0.15 M Tris-Hcl buffer. A part of homogenate after precipitating proteins with trichloroacetic acid (TCA) was used for analysis.

### Cytochrome P450 and Cytochrome b5

Cytochrome P450 was determined using the carbon monoxide difference spectra. Both cytochrome P450 and cytochrome b5 content were assayed in microsomal suspension by the method of Omura and Sato [81], using an absorption coefficient of 91 and 185 cm2 M^-1^ m^-1^, respectively.

### Glutathione S-transferase

Glutathione S-transferase (GST) activity was determined spectrophotometrically at 37° C as described earlier [82]. Briefly, the reaction mixture (1ml) contained 0.334 ml of 100 mM phosphate buffer (pH 6.5), 0.033 ml of 30 mM CDNB and 0.033 ml of 30 mM of reduced glutathione. After pre incubating the reaction mixture for 2 minutes, the reaction was started by adding 0.01 ml of diluted homogenate and the absorbance was followed for 3 minutes at 340 nm. The specific activity of GST is expressed as μ moles of GSH-CDNB conjugate formed/min/mg protein using extinction co-efficient of 9.6 mM^–1^ cm^–1^.

### Statistical analysis

The data represents mean ± STD. The Dunnett’s multiple comparison test was used for the analysis. *P* value < 0.001, <0.01, <0.05 was considered significant.

## CONCLUSIONS

On the basis of above finding it is concluded that FEO exhibited *in vitro* anti-melanoma activity by reducing the viability in B16-F10 cell, FM94 cells and not in HNEM cells by the induction of apoptosis *via* caspase signaling and MCL-1 dependent pathway. Furthermore, FEO can reduce tumour size in C57BL/6 mice melanoma tumour model. FEO can be most effective drug for prevention of hepatic injury along with improved hematological biochemical parameters, liver histology, phase I and phase II drug metabolizing enzymes.
