# Correction: Frankincense essential oil suppresses melanoma cancer through down regulation of Bcl-2/Bax cascade signaling and ameliorates heptotoxicity via phase I and II drug metabolizing enzymes

**DOI:** 10.18632/oncotarget.27540

**Published:** 2020-06-09

**Authors:** Faruck L. Hakkim, Hamid A. Bakshi, Shabia Khan, Mohamed M. Nasef, Rabia Farzand, Smitha Sam, Luay Rashan, Mohammed S. Al-Baloshi, Sidgi Syed Anwar Abdo Hasson, Ali Al Jabri, Paul A. McCarron, Murtaza M. Tambuwala

**Affiliations:** ^1^ Frankincense Biodiversity Unit, Research Center, Dhofar University, Salalah, Oman; ^2^ Department of Pharmacy, School of Applied Sciences, University of Huddersfield, Queensgate, Huddersfield, United Kingdom; ^3^ Department of Pathobiological Sciences, School of Veterinary Medicine, Louisiana State University, Baton Rouge, LA, USA; ^4^ Department of Clinical and Pharmaceutical Sciences, University of Hertfordshire, Hertfordshire, United Kingdom; ^5^ Chemotherapy Unit, St. Jude Clinics-Center for Cancer Treatment, Pathanamthitta, Kerala, India; ^6^ Department of Mathematics and Sciences, College of Arts and Applied Sciences, Dhofar University, Salalah, Oman; ^7^ Department of Microbiology and Immunology, College of Medicine and Health Sciences, Sultan Qaboos University, Al-Khoud, Muscat, Oman; ^8^ School of Pharmacy and Pharmaceutical Science, SAAD Centre for Pharmacy and Diabetes, Ulster University, Coleraine, County Londonderry, Northern Ireland, United Kingdom

**This article has been corrected:** This article has been corrected: Due to errors during image selection, identical images were used for panels D and E in Figure 1D. In Figure 7A, the brain images for ‘healthy’ and ‘treated’ are also accidental duplicates. Additionally, the name of the 4th author in the listing has been updated, correcting the name as follows:

**Mohamed M. Nasef^2^**

The authors declare that these corrections do not change the results or conclusions of this paper.

Original article: Oncotarget. 2019; 10:3472–3490. 3472-3490. https://doi.org/10.18632/oncotarget.26930

**Figure 1:**
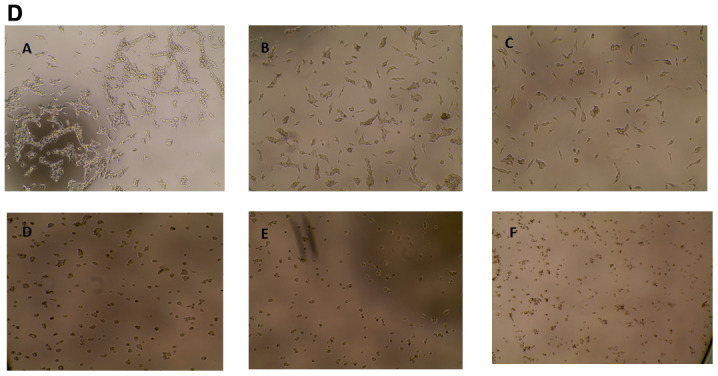
Cytotoxicity of FEO on B16-F10, FM94 and HNEM cells.

(**D**) FM94 cells treated with FEO (A-Untreated cells; B-3 μg/ml; C-5 μg/ml; D-7 μg/ml; E-10 μg/ml; F- Dox 5 ug/ml) for 24 h and morphological image was photographed by EVOS image analyser; E: HNEM cells treated with FEO (A-Untreated cells; B-3 μg/ml; C-5 μg/ml; D-7 μg/ml; E-10 μg/ml; F- Dox 5 ug/ml) for 24 h and morphological image was photographed by EVOS image analyser. Data presented as mean ± SD of triplicates of three independent experiments. ^*^Represents significant difference at *p* < 0.05 compared with control. **Represents significant difference at *p* < 0.01 compared with control. ^***^Represents significant difference at *p* < 0.001 compared with control. NS: Non significant. Scale bar indicates 10 um.

**Figure 7:**
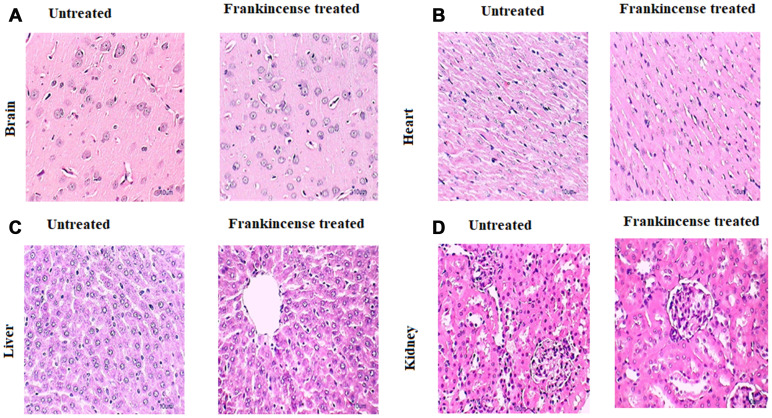
*In vivo* of toxicity of FEO on major organs: Mice were treated with FEO (1200 mg/kg body weight).

At the end of experimental period major organs such as brain, heart, liver and kidney were excised and stained with hemotoxylin and eosin. Scale bar indicates 10 μm.

